# A Keyframe Extraction Method for Assembly Line Operation Videos Based on Optical Flow Estimation and ORB Features

**DOI:** 10.3390/s25092677

**Published:** 2025-04-23

**Authors:** Xiaoyu Gao, Hua Xiang, Tongxi Wang, Wei Zhan, Mengxue Xie, Lingxuan Zhang, Muyu Lin

**Affiliations:** School of Computer Science, Yangtze University, Jingzhou 434023, China; 2023710706@yangtzeu.edu.cn (X.G.);

**Keywords:** keyframe extraction, assembly line operation videos, optical flow estimation, ORB features, bag-of-visual-words model, k-means++ clustering

## Abstract

In modern manufacturing, cameras are widely used to record the full workflow of assembly line workers, enabling video-based operational analysis and management. However, these recordings are often excessively long, leading to high storage demands and inefficient processing. Existing keyframe extraction methods typically apply uniform strategies across all frames, which are ineffective in detecting subtle movements. To address this, we propose a keyframe extraction method tailored for assembly line videos, combining optical flow estimation with ORB-based visual features. Our approach adapts extraction strategies to actions with different motion amplitudes. Each video frame is first encoded into a feature vector using the ORB algorithm and a bag-of-visual-words model. Optical flow is then calculated using the DIS algorithm, allowing frames to be categorized by motion intensity. Adjacent frames within the same category are grouped, and the appropriate number of clusters, *k*, is determined based on the group’s characteristics. Keyframes are finally selected via k-means++ clustering within each group. The experimental results show that our method achieves a recall rate of 85.2%, with over 90% recall for actions involving minimal movement. Moreover, the method processes an average of 274 frames per second. These results highlight the method’s effectiveness in identifying subtle actions, reducing redundant content, and delivering high accuracy with efficient performance.

## 1. Introduction

Despite advancements in automation, many assembly line processes in industrial production still rely heavily on manual labor. This reliance often results in inefficiencies, with one of the primary causes being non-standardized operational behaviors among workers. As a result, standardizing and managing these manual operations has become critical to improving overall productivity.

To address this issue, many manufacturing enterprises have adopted real-time video monitoring systems on production floors. These systems serve multiple functions, including emergency detection, worker safety monitoring, and human activity analysis [1]. Beyond ensuring safety, they also enable the evaluation and optimization of operational workflows [2], support data-driven production planning, and ultimately enhance product quality and manufacturing efficiency [3]. However, continuous video surveillance generates vast amounts of redundant footage [4], leading to excessive storage demands and increased costs associated with data transmission, action recognition, and semantic analysis [5]. Direct processing of such large-scale video data is both inefficient and resource-intensive. A more efficient solution is to extract keyframes and replace the original video with a representative subset, thereby reducing storage and computational overhead while retaining essential information.

Developing a robust keyframe extraction method is therefore essential for summarizing large-scale video data from assembly lines. Such a method would enable efficient anomaly detection, streamline quality control, and facilitate timely decision-making by significantly lowering data volume while preserving critical content.

Keyframe extraction, a fundamental task in computer vision, aims to identify frames that capture the core content and structure of a video. By reducing data redundancy, it improves the efficiency of video storage, analysis, and retrieval. In recent years, extensive research has led to the development of diverse keyframe extraction techniques, which can be broadly classified into five categories: image feature-based methods [6], shot detection-based methods [7,8,9], motion-based methods [10,11], deep learning-based methods [12,13,14], and clustering-based methods [15,16,17].

(1) Image Feature-Based Methods

These methods identify keyframes by analyzing low-level image features such as color, texture, and brightness. Zhang et al. [18] proposed calculating color histogram differences between consecutive frames to select representative keyframes. Lai et al. [19] enhanced this approach by incorporating motion and saliency features. Typically, these methods compare adjacent frames based on predefined thresholds. However, their performance is sensitive to threshold selection, and they often neglect global frame relationships, resulting in redundant keyframes and low computational efficiency.

(2) Shot Detection-Based Methods

Shot detection methods extract keyframes by identifying scene changes. For example, Jadhav et al. [20] used statistical color moments to detect shot boundaries, while Hannane et al. [21] applied SIFT-based histograms for boundary detection. Although effective in traditional video content, these methods are less suitable for assembly line videos, which often lack clear scene transitions due to repetitive and highly similar frames.

(3) Motion Information-Based Methods

Recent approaches have leveraged motion information to improve keyframe extraction. Zhang et al. [22] used a multi-population genetic algorithm to extract keyframes from human motion capture data, aiming to reduce reconstruction error and improve compression. Xia et al. [23] proposed a Joint Kernel Sparse Representation model that accounts for motion sparsity and manifold structure. While effective, motion-based methods often involve complex computations, limiting their applicability in real-time industrial settings.

(4) Deep Learning-Based Methods

With the rise of deep learning, numerous studies have explored neural network-based keyframe extraction. Kızıltepe et al. [24] combined CNNs and RNNs to identify salient regions in video frames and select representative keyframes. Others, such as Dhiman et al. [25], used pre-trained models like ResNet151V2 to extract semantic features and applied unsupervised clustering for keyframe selection. While these methods achieve high accuracy, they require extensive training data and manual annotations, resulting in high development costs.

(5) Clustering-Based Methods

Clustering approaches group similar frames and select representative frames from each cluster [26]. Furini et al. [27] introduced STIMO, which uses HSV-based clustering for real-time performance. De Avila et al. [28] proposed a k-means-based method leveraging color features. However, due to the cyclical and repetitive nature of assembly line tasks, global clustering may lead to uneven keyframe distribution, with excessive redundancy in some segments and inadequate representation in others.

Moreover, most existing methods apply a uniform extraction strategy across all frames, failing to account for the varying motion scales in assembly line activities. Large-scale movements, such as arm swings, require fewer keyframes, yet their pronounced differences between frames often result in overrepresentation. In contrast, fine-grained actions—such as finger movements—require more detailed sampling but are frequently underrepresented due to frame similarity. Notably, such fine actions are of particular interest to supervisors, as they often involve direct product manipulation.

To overcome these limitations, we propose a keyframe extraction method tailored for assembly line operation videos, combining optical flow estimation with ORB-based feature encoding. Each frame is first encoded using the ORB (Oriented FAST and Rotated BRIEF) algorithm [29] and a bag-of-visual-words model [30]. Optical flow is then computed using the DIS (Dense Inverse Search) algorithm [31] to estimate motion intensity. Based on optical flow values, frames are categorized and temporally grouped. The number of clusters, *k*, for each group is determined by both the motion category and frame count. Finally, keyframes are extracted using k-means++ clustering on the corresponding feature vectors.

The innovations and contributions of this paper are summarized as follows:

- Applying keyframe extraction techniques to industrial assembly line scenarios can significantly reduce storage and computational resource consumption while enhancing video processing efficiency. This approach supports anomaly detection and quality control in production processes, contributing to more efficient and reliable industrial operations.
- The optical flow field is used to simulate the trajectory of assembly line actions. The mean magnitude of the optical flow vectors for all pixel points from the previous frame to the current frame is calculated as the optical flow value of the current frame. The optical flow value is then used to measure the motion amplitude in the video.
- A method is designed to calculate the value of *k* in k-means++ clustering based on optical flow categories. This method enables the adoption of appropriate keyframe extraction strategies for actions of different amplitudes in assembly line operation videos. For video frame categories with small optical flow values, a higher compression rate is achieved, effectively capturing small-scale actions and targeting assembly line motions more precisely.
- Video frames are grouped based on their categories and sequence numbers, with adjacent frames of the same category grouped together. Clustering is performed separately for each group, preventing keyframes from being overly concentrated in certain video segments. Furthermore, frames within the same group are generally similar, and clustering similar frames helps to reduce video frame redundancy more effectively.

The remainder of this paper is organized as follows. Section 2 describes the detailed process of the proposed method. Section 3 presents the experimental results and discusses them. Finally, Section 4 summarizes the work conducted in this study.

## 2. Methodology

**Figure 1:**
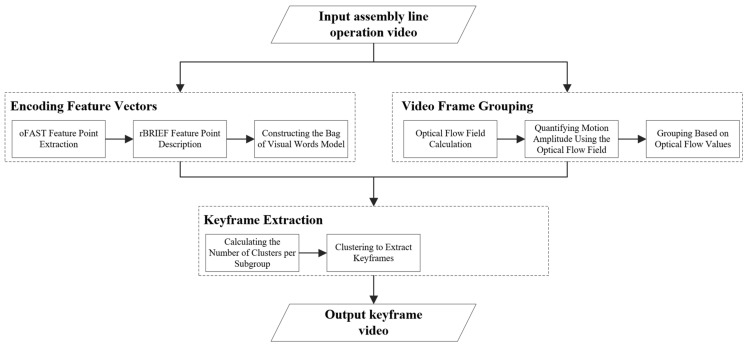
Flowchart of the proposed method.

The workflow of the proposed method is shown in Figure 1. First, an assembly line operation video is input. Next, the ORB algorithm is used to extract feature points from the video frames and generate descriptors for these feature points. Based on the descriptors, a bag-of-visual-words model is constructed, encoding each video frame into a feature vector. Then, the DIS algorithm is applied to calculate the optical flow value for each video frame. Using these optical flow values, the video frames are clustered and categorized into several classes. The frames are further grouped based on their categories and sequence numbers. Subsequently, the number of clusters, *k*, is determined for each group. The k-means++ clustering algorithm is then applied to the feature vectors of each group, selecting the frame closest to the cluster center in each cluster as the keyframe. Finally, all keyframes are arranged in ascending order according to their sequence numbers, forming a new video, which is then output.

Although feature vector encoding and video frame grouping are temporally independent stages in our method, they work in a complementary and coordinated manner within the overall framework. First, each frame undergoes local feature extraction using the ORB algorithm. The extracted features are then encoded into fixed-length vectors using a bag-of-visual-words model, providing a compact representation of visual information. This step enhances the accuracy and representativeness of subsequent keyframe selection.

Next, the DIS optical flow algorithm is applied to compute motion vectors for each frame. These values are used to quantify motion intensity, based on which frames are categorized and grouped according to both temporal order and motion characteristics. While the grouping process does not directly interact with the feature vectors, it enables adaptive treatment of frames with varying motion intensity. This helps prevent suboptimal keyframe distributions and avoids the loss of subtle yet important movements in assembly line videos due to overly rigid selection strategies.

In the keyframe extraction stage, the two components converge. The previously computed feature vectors are used to perform k-means++ clustering within each frame group. The integration of these two components ensures that the selected keyframes are representative in both temporal and visual dimensions, thereby enhancing their overall quality.

The following sections provide a more detailed description of the method’s workflow.

### 2.1. Encoding Feature Vectors

#### 2.1.1. oFAST Feature Point Extraction

The ORB (Oriented FAST and Rotated BRIEF) algorithm is an improved and optimized version of the FAST [32] (Features from Accelerated Segment Test) corner detection and BRIEF [33] (Binary Robust Independent Elementary Features) descriptor [29]. ORB achieves scale invariance by extracting corners at each layer of an image pyramid. The process of extracting descriptors for video frames using the ORB algorithm involves two steps: first, oFAST (Oriented FAST) is used to extract directional feature points, and then rBRIEF (Rotated BRIEF) descriptors are generated for these feature points.

This study employs the ORB algorithm, primarily due to its high computational efficiency and robustness to lighting variations. First, ORB utilizes FAST keypoint detection and BRIEF binary descriptors for rapid feature extraction, making it well-suited for real-time processing. Second, the BRIEF descriptor relies on the relative differences in pixel intensity within local regions rather than absolute intensity values, effectively reducing the impact of lighting variations on feature matching.

**Figure 2:**
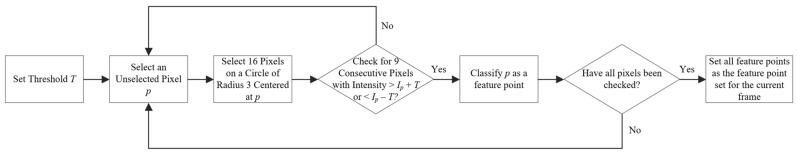
Flowchart of the FAST algorithm.

**Figure 3:**
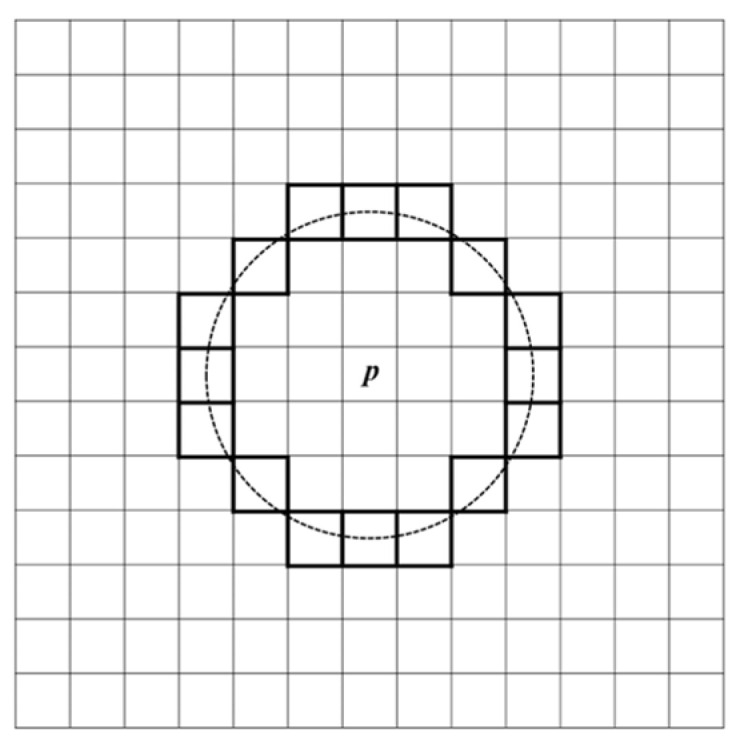
Illustration of FAST feature point extraction.

The FAST algorithm determines whether a pixel is a feature point by comparing its intensity value with those of its surrounding pixels. The workflow of the FAST algorithm is shown in Figure 2. First, a threshold *T* is set. For each pixel *p* (with intensity value *I_p_*) in each layer of the scale-space pyramid of the video frame, 16 pixels on a circle with a radius of 3 centered at *p* are selected (as shown in Figure 3). If there are at least 9 consecutive pixels in this circle whose intensity values are either greater than *I_p_* + *T* or less than *I_p_* − *T*, then *p* is classified as a feature point. All detected feature points are collected as the feature point set for the current frame.

**Figure 4:**
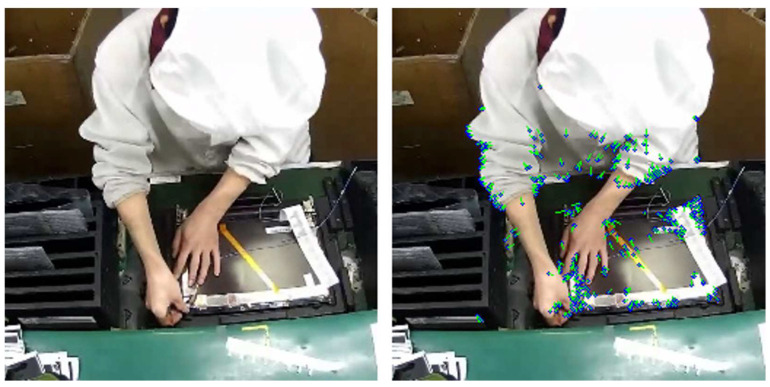
Illustration of oFAST feature points.

The ORB algorithm employs oFAST for feature point detection. Based on FAST, oFAST uses the intensity centroid method to assign a direction to feature points, thereby enhancing robustness to image rotation. The feature points detected by oFAST are shown in Figure 4, where the blue dots represent the feature points, and the green lines indicate their directions.

The specific steps for assigning directions to feature points in the oFAST algorithm are as follows:

(1) A neighborhood moment is selected with the feature point as the center. The neighborhood moment is defined as follows:

(1) $$m_{pq}=\underset{x,y}{\sum }x^{p}y^{q}I\left(x,y\right)$$

Here, *I*(*x*, *y*) represents the pixel value at the coordinates (*x*, *y*), and *p* and *q* are the orders of the moment.

(2) The centroid of the neighborhood moment is calculated as follows:

(2) $$C=\left(\frac{m_{10}}{m_{00}},\frac{m_{01}}{m_{00}}\right)$$

(3) A vector is constructed from the feature point to the centroid of the neighborhood moment. The direction of this vector is assigned as the direction of the feature point. The direction is calculated using the following formula:

(3) $$\theta =\arctan\left(m_{01},m_{10}\right)$$

#### 2.1.2. rBRIEF Feature Point Description

The ORB algorithm generates rBRIEF descriptors for the feature points detected by oFAST. rBRIEF, based on BRIEF, introduces rotation invariance to the descriptors. The steps for rBRIEF to generate descriptors for the feature points extracted by oFAST are as follows:

To reduce the impact of noise, Gaussian filtering is first applied to the image. Then, a 31 × 31 neighborhood is selected around each feature point as the center. Within this neighborhood, 256 pixel pairs are selected. For each pixel pair (*x_i_*, *y_i_*), a binary test is performed. The test values of the 256 pixel pairs form a 256-dimensional descriptor as follows:

(4) $$f_{n}(p)=\underset{1\leq i\leq n}{\sum }2^{i-1}\tau \left(p;x_{i},y_{i}\right)$$

To provide the descriptor with a certain degree of rotational invariance, rBRIEF rotates the point pairs using the feature point direction *θ* determined by oFAST. The set of 256 point pairs is represented by a matrix *S* as follows:

(5) $$S=\left(\begin{array}{c} x_{1},\ldots ,x_{n}\\ y_{1},\ldots ,y_{n} \end{array}\right)$$

The rotation matrix *R_θ_* is constructed based on the feature point direction *θ* as follows:

(6) $$\left.R_{\theta }=\left(\begin{array}{cc} \cos\theta & -\sin\theta \\ \sin\theta & \cos\theta \end{array}\right.\right)$$

The matrix *S* is rotated to obtain the matrix *S_θ_* as follows:

(7) $$S_{\theta }=R_{\theta }S$$

Finally, the rBRIEF descriptor with rotational invariance is generated as follows:

(8) $$g_{n}\left(p,\theta \right)=f_{n}\left(p\right)|\left(x_{i},y_{i}\right)\in S_{\theta }$$

#### 2.1.3. Constructing the Bag-of-Visual-Words Model

The bag-of-visual-words (BoVW) model [30] is a feature encoding method that classifies image feature point descriptors into visual words using clustering algorithms. It then counts the occurrences of each visual word in an image and encodes the image as a vector. The specific steps for using the BoVW model to encode video frames into feature vectors in this study are as follows:

(1) Perform k-means++ clustering on the descriptors of all video frames *X* = {*x*_1_, *x*_2_, …, *x_N_*}, resulting in *k* cluster centers *C* = {*c*_1_, *c*_2_, …, *c**_k_***}. Each cluster center *c_i_* is referred to as a visual word, and the set *C* is called the visual vocabulary.

(2) Given a video frame *F* with a descriptor set *D* = {*d*_1_, *d*_2_, …, *d_n_*}, assign each descriptor to the visual word with the smallest Euclidean distance. Construct a vector *V* = (*v*_1_, *v*_2_, …, *v_k_*), where *v_i_* represents the number of descriptors from frame F assigned to the visual word *c_i_* ∈ *C*.

(3) Perform L2 normalization on the vector *V* = (*v*_1_, *v*_2_, …, *v_k_*):

(9) $$V_{\mathrm{norm}}=\frac{V}{∥V{∥}_{2}}$$

(10) $$∥V{∥}_{2}=\sqrt{\overset{k}{\underset{i=1}{\sum }}v_{i}^{2}}$$

### 2.2. Video Frame Grouping

#### 2.2.1. Optical Flow Field Calculation

In the field of computer vision, optical flow algorithms are widely used to estimate the motion trajectories of pixels in an image sequence. The proposed method employs the optical flow field to simulate the trajectories of assembly line actions. Traditional optical flow methods have high time complexity and low computational efficiency, making them unsuitable for applications requiring real-time processing. To address this issue, Kroeger et al. [31] proposed an optical flow algorithm called DIS (Dense Inverse Search), which effectively balances computation speed and accuracy, significantly improving the efficiency of optical flow calculation. The DIS algorithm efficiently determines block correspondences between images using inverse search, then creates a dense displacement field through multi-scale block aggregation, and finally applies variational refinement to enhance accuracy. The specific steps are as follows:

(1) Grid Creation: A uniform grid is constructed over the image domain to initialize image blocks. The density and number of blocks *N_s_* are determined by the parameter *θ_ov_* ∈ [0, 1), which specifies the overlap ratio between adjacent blocks. A value of *θ_ov_* = 0 indicates no overlap.

(2) Initialization: For the first iteration (*s* = *θ_ss_*), the optical flow vector in each block is initialized to zero. For subsequent scales *s*, the displacement of each block is initialized based on the flow from the previous scale.

(3) Inverse Search: The goal of inverse search is to independently calculate the displacement of all blocks by minimizing the squared difference between blocks using gradient descent. The formula is as follows:

(11) $$u={\mathrm{argmin}}_{u\prime }\sum_{x}{\left[I_{t+1}(x+u\prime )-T(x)\right]}^{2}$$

In this formula, *u* is the displacement vector, *I_t+_*_1_ represents the next frame image, *T* is the current template block, and *x* denotes the pixel coordinates within the block.

(4) Densification: For all blocks overlapping at pixel *x*, the dense flow field U_s_ for each pixel *x* is created by computing the weighted average of the displacement estimates:

(12) $$U_{s}\left(x\right)=\frac{1}{Z}\overset{N_{s}}{\underset{i}{\sum }}\frac{\lambda_{i,x}}{\max(1,\|\;d_{i}(x){\|}_{2})}\cdot u_{i}$$

(13) $$Z=\frac{\sum_{i}\lambda_{i,x}}{\max(1,\|\;d_{i}(x){\|}_{2})}$$

(14) $$d_{i}\left(x\right)=I_{t+1}\left(x+u_{i}\right)-T\left(x\right)$$

In this formula, the indicator λ_i,x_ equals 1 when block *i* overlaps with position *x* in the reference image; otherwise, *λ_i,x_* equals 0. The term *d_i_*(*x*) represents the intensity difference between the template block and the deformed image at this pixel, *u_i_* denotes the displacement estimate of block *i*, and *Z* represents normalization.

(5) Variational Refinement: After completing the densification step, variational refinement is used to further improve the accuracy of the optical flow field. This process is achieved by minimizing an energy function that includes a data term and a smoothness term. The formula for the energy function is as follows:

(15) $$E\left(U\right)=\int_{\Omega }\sigma \Psi (E_{l})+\gamma \Psi (E_{G})+\alpha \Psi (E_{S})dx$$

Here, U denotes the optical flow field over the image domain *Ω*, and *ψ*() is a robust penalty function designed to mitigate the influence of outliers. The weighting factors *σ*, *γ*, and *α* balance the contributions of three distinct energy terms. The brightness consistency term *E_I_* enforces photometric similarity between corresponding pixels in consecutive frames. The gradient consistency term *E_G_* preserves the local image gradient structures, thereby accounting for illumination variations and enhancing edge alignment. Finally, the smoothness term *E_S_* regularizes the optical flow field by promoting spatial coherence and reducing abrupt variations. Minimizing this energy functional refines the initial optical flow estimate, achieving an optimal compromise between data fidelity and spatial regularity, and ultimately leading to a more robust and efficient optical flow estimation process.

**Figure 5:**
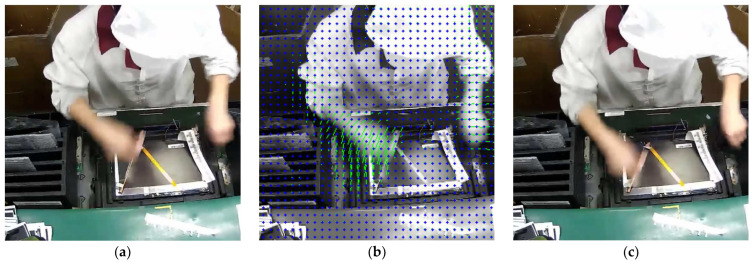
Illustration of the dense optical flow field. (**a**) The current frame. (**b**) The optical flow field from the current frame to the next frame. (**c**) The next frame.

Through the above steps, a dense optical flow field is obtained, describing the displacement of each pixel from one frame to the next. The dense optical flow field is shown in Figure 5, where blue dots represent pixel points, and green lines represent optical flow. The dense optical flow field effectively simulates the trajectory of assembly line actions.

#### 2.2.2. Quantifying Motion Amplitude Using the Optical Flow Field

Motion amplitude refers to the physical spatial range covered by a person or object during a specific movement. The dense optical flow field describes the displacement of each pixel from one frame to the next. In this study, the optical flow value computed from the dense optical flow field is used to quantify the magnitude of motion amplitude. From the dense optical flow field, the optical flow vector (*x_i_*, *y_i_*) for each pixel can be obtained, where *x_i_* and *y_i_* represent the horizontal and vertical displacements of pixel *i*, respectively. The steps for calculating the optical flow value of a frame based on the dense optical flow field are as follows:

(1) Calculate the magnitude of the optical flow vector for each pixel from the previous frame to the current frame:

(16) $$∥{\vec{v}}_{i}∥=\sqrt{x_{i}^{2}+y_{i}^{2}}$$

(2) Compute the average magnitude of the optical flow vectors. Sum the magnitudes of the optical flow vectors for all pixels from the previous frame to the current frame, then divide the sum by the total number of pixels. This value is used as the optical flow value for the current frame:

(17) $$value=\frac{1}{N}\overset{N}{\underset{i=1}{\sum }}∥{\vec{v}}_{i}∥$$

The optical flow value of a frame reflects the overall displacement of pixels from the previous frame to the current frame. In this study, the optical flow value of a frame is used to measure the magnitude of motion amplitude in a video segment. A larger optical flow value indicates greater motion amplitude at that moment.

#### 2.2.3. Grouping Based on Optical Flow Values

**Figure 6:**
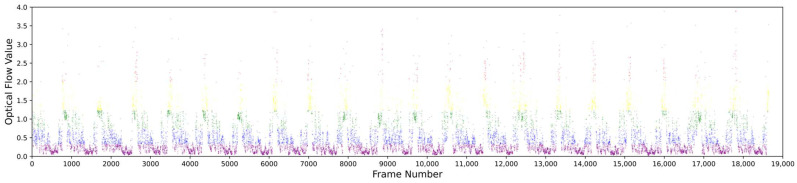
Illustration of clustering results.

**Figure 7:**
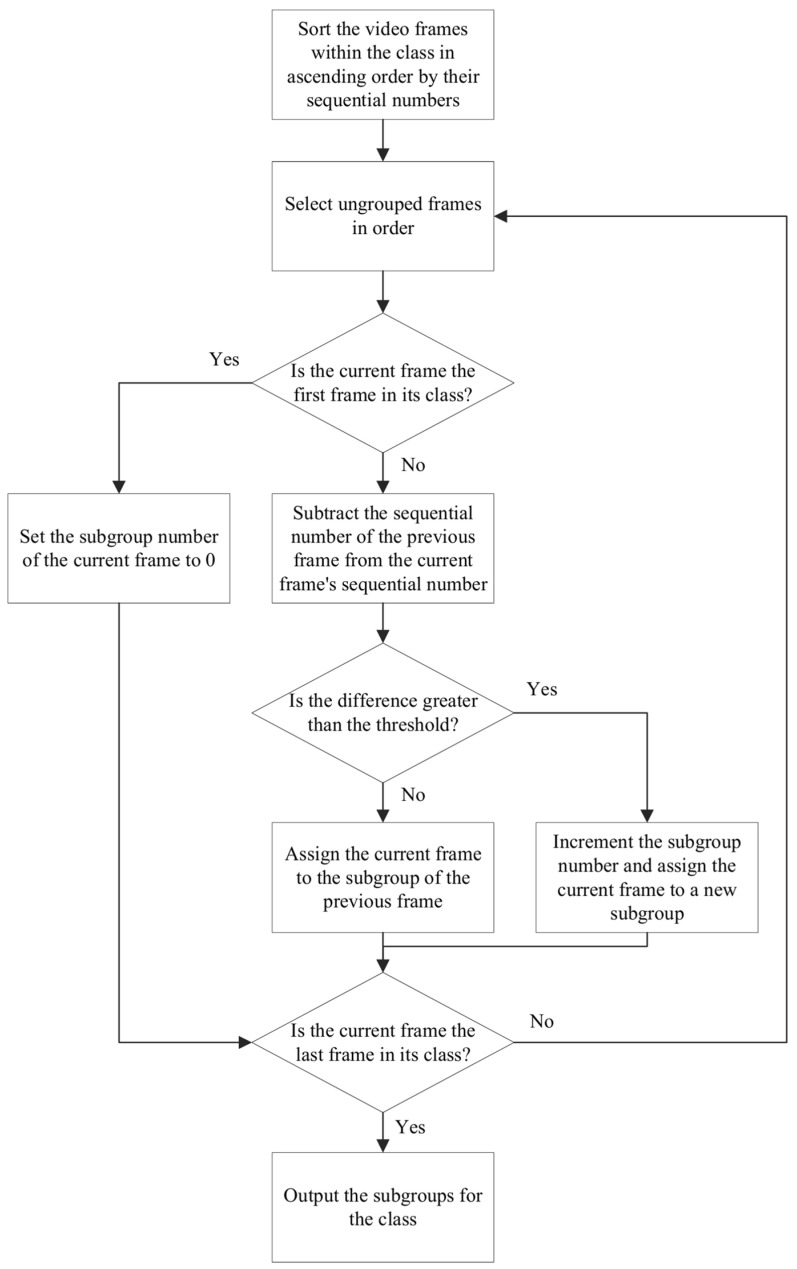
Flowchart of video frame grouping.

To adopt appropriate keyframe extraction strategies for video segments with different motion amplitudes and to prevent keyframes from being overly concentrated in certain segments, this method groups video frames based on their optical flow values:

(1) Assign sequential numbers to the video frames in the order they appear in the original video, starting from 1.

(2) Perform k-means++ clustering on the video frames based on their optical flow values, dividing the frames into several classes. Figure 6 illustrates the results of clustering video frames using the k-means++ algorithm based on optical flow values. In our approach, frames are grouped into five categories, each represented by a different color, where frames within the same cluster exhibit similar motion intensities. The figure reveals a quasi-periodic pattern in the distribution of optical flow across frames in the assembly line video. This pattern arises from the repetitive nature of workers’ actions, where each cycle of operation follows a similar structure, producing recurring motion characteristics. Such periodicity presents a challenge for keyframe extraction. Performing global clustering on the entire video may lead to an overconcentration of keyframes within certain repetitive segments, resulting in redundancy, while other segments may lack sufficient keyframes to capture important actions—ultimately compromising representational completeness. To address this, we introduce a grouping strategy that segments adjacent frames within the same cluster into coherent groups. This ensures that keyframes are more evenly distributed across different action cycles, leading to a more comprehensive and balanced summarization.

(3) To group video frames based on their class and sequential numbers, the frames within each class are first sorted in ascending order by their sequential numbers. The first frame in each class is assigned to subgroup 0 by default. For each subsequent frame, the difference between its sequential number and that of the previous frame is calculated. If the difference is less than or equal to a predefined threshold, the current frame is assigned to the same subgroup as the previous frame. Otherwise, the subgroup number is incremented, and the current frame is assigned to a new subgroup. This process is repeated until all frames within the class are grouped, and the subgroups for each class are then output. The grouping process is illustrated in Figure 7.

### 2.3. Keyframe Extraction

#### 2.3.1. Calculating the Number of Clusters per Subgroup

To better capture small-scale assembly line actions, this method applies different compression rates to video frames of different categories. Video frame classes with smaller optical flow values are assigned higher compression rates, which are controlled by the number of clusters in each subgroup. The steps for calculating the number of clusters per subgroup are as follows:

(1) Based on the center values of each class obtained during the classification of video frames, calculate the proportion of each class’s center value relative to the sum of the center values of all classes:

(18) $$P_{i}=\frac{C_{i}}{\sum_{j=1}^{n}C_{j}}$$

Here, *C_i_* represents the center value of the i-th class, and *n* is the total number of classes.

(2) Calculate the number of clusters for each subgroup as follows:

(19) $$k=\frac{L}{a+bP_{i}}$$

In the formula, *L* represents the number of frames in a subgroup, while 1/(*a* + *bP_i_*) determines the compression rate for each subgroup. The constant *a* ensures a baseline compression level across all subgroups, whereas the constant *b* is used to adjust the compression rate. A larger *b* results in lower compression rates for video frames with higher optical flow values, thereby increasing the difference in compression rate across different frame categories. This formula enables the proposed method to apply an adaptive compression rate to actions of varying magnitudes in assembly line operation videos. It ensures a higher keyframe extraction rate for subtle movements, effectively capturing finer details of assembly line operations.

#### 2.3.2. Clustering to Extract Keyframes

**Figure 8:**
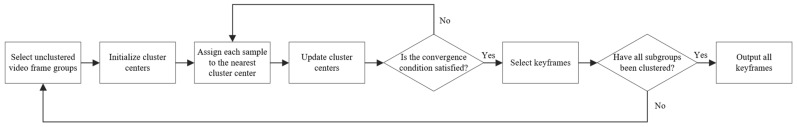
Flowchart of cluster-based keyframe extraction.

k-means++ is an improved version of the k-means algorithm that optimizes the initialization process for cluster centers, enhancing clustering performance. While k-means randomly selects cluster centers, k-means++ selects them based on the principle that the initial cluster centers should be as far apart as possible. The process works as follows: First, one center point is chosen randomly. Then, for each sample point, the probability of it becoming the next cluster center is calculated based on its distance to the existing cluster centers—the greater the distance, the higher the probability. The next cluster center is then selected based on this probability. The workflow for using k-means++ clustering on feature vectors to extract keyframes from video frame subgroups is illustrated in Figure 8:

(1) Input the feature vector set of the video frame subgroup and the number of clusters *k_i_* for the subgroup.
(2) Initialize the cluster centers:
  (a) Randomly select one sample point as the first cluster center.
  (b) Use the square of the Euclidean distance as the measure of distance between sample points. Calculate the distance *D*(*s_i_*) from each unselected sample point *s_i_* to the nearest cluster center. In n-dimensional space, the square of the Euclidean distance between point *b*(*b*_1_, *b*_2_, …, *b_n_*) and point *c*(*c*_1_, *c*_2_, …, *c_n_*) is calculated using the following formula:
    (20) $$d\left(b,c\right)=\overset{n}{\underset{i=1}{\sum }}{\left(b_{i}-c_{i}\right)}^{2}$$
  (c) Calculate the selection probability for each unselected sample point *s_i_*:
    (21) $$P\left(s_{i}\right)=\frac{D(s_{i})}{\sum_{s_{j}\in S}D(s_{j})}$$
  (d) Select the next cluster center based on the calculated probabilities.
  (e) Repeat steps (b), (c), and (d) until *k_i_* cluster centers are selected.
(3) Calculate the Euclidean distance from each sample point to each cluster center, and assign the sample point to the cluster of the nearest cluster center.
(4) After all sample points have been assigned to their respective clusters, update each cluster center. The new cluster center is the mean of the sample points in that cluster.
(5) Repeat steps 3 and 4 until the convergence condition is met: either the change in cluster centers is less than a predefined threshold (1 × 10^−4^) or the maximum number of iterations (300) is reached.
(6) Select the frame corresponding to the sample point closest to the cluster center in each cluster as a keyframe.
(7) Repeat the above steps until the keyframes for all subgroups have been computed.

## 3. Experimental Results and Discussion

### 3.1. Dataset

**Figure 9:**
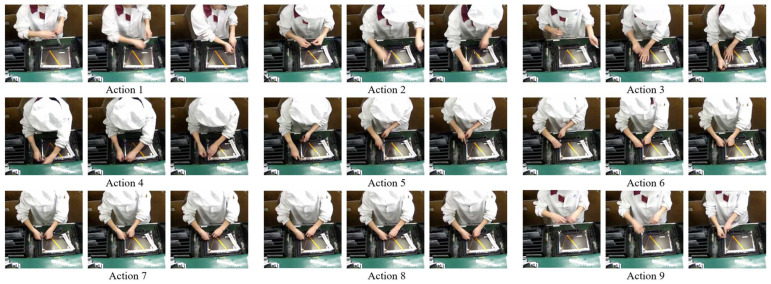
Illustration of pipeline actions.

The dataset used in this study was collected from surveillance footage of real worker operations on an industrial assembly line. The videos were recorded using Hikvision DS-2CD2051-1 network cameras (manufactured by Hikvision, Hangzhou, China), with an MP4 format, a frame rate of 24 frames per second, and a resolution of 720 × 720, comprising a total of 18,648 frames. The dataset captures 21 cycles of production line activities, with each cycle following the same sequence of actions, as illustrated in Figure 9. A standard production cycle consists of nine distinct actions, sequentially numbered from 1 to 9. To ensure that the extracted frames accurately represent each cycle’s key operational states, keyframes were selected for every cycle.

### 3.2. Implementation Details

(1) Running Environment: The experimental environment for this study is shown in Table 1. All computations were performed on a CPU.

(2) Resolution Adjustment: To reduce computational load and meet real-time requirements, the video resolution was reduced from 720 × 720 to 256 × 256 during processing.

(3) Parameter Settings: In this study, up to 100 feature points are extracted from each video frame, which are then encoded into a 32-dimensional feature vector. The frames are categorized into five classes, with a grouping threshold of 48. In Equation (20), the constants *a* and *b* are set to 15 and 30, respectively. These parameter values should be adjusted based on the characteristics of the dataset.

### 3.3. Evaluation Metrics

(1) Precision (*P*), Recall (*R*), and F-score (*F*):

Precision (*P*), recall (*R*), and F-score (*F*) are commonly used metrics in the field of video keyframe extraction to evaluate the performance of methods:

(22) $$P=\frac{{\mathrm{Num}}_{mf}}{{\mathrm{Num}}_{vs}}$$

(23) $$R=\frac{{\mathrm{Num}}_{mf}}{{\mathrm{Num}}_{gt}}$$

Here, *Num_mf_* represents the number of frames matched between the keyframes extracted by the method and the ground truth labels. *Num_vs_* is the total number of keyframes extracted by the method, and *Num_gt_* is the total number of frames in the ground truth labels [34].

In the field of keyframe extraction, recall is commonly used to measure the proportion of keyframes extracted by the method that match the ground truth labels. However, this study focuses on the assembly line actions of workers. A single assembly line action typically lasts 2 to 4 s, resulting in tens of frames to represent one action. For small-scale hand movements, the differences between consecutive frames are minimal, making it difficult to determine which frames are best suited as ground truth labels. The selection of ground truth labels is thus prone to subjectivity, leading to issues in recall calculation. Moreover, if all frames representing an assembly line action are simply considered as keyframes, the value of *Num_gt_* becomes excessively large, causing *R* to be very small and reducing the effectiveness of recall as an evaluation metric. Additionally, this study prioritizes the assembly line actions of workers, aiming to minimize the number of extracted keyframes while ensuring that the extracted keyframes can represent as many complete assembly line actions as possible. Therefore, in this study, recall is calculated by considering a “complete assembly line action” as a positive sample. Recall is defined as the ratio of the number of complete assembly line actions represented by the extracted keyframes to the total number of assembly line actions in the video. The method for calculating the number of complete assembly line actions will be introduced next.

As shown in Figure 9, each type of pipeline action is represented by three images, illustrating the initial, intermediate, and final states of the action. These images are referred to as the action’s initial frame, process frame, and end frame, respectively. The calculation method for the number of complete pipeline actions in this study is as follows: if the set of keyframes extracted by a method contains the initial frame, process frame, and end frame for *n* pipeline actions, then the number of complete pipeline actions demonstrated by the extracted keyframes is *n*.

The formulas for calculating the precision (*P*), recall (*R*), and F-score (*F*) in this study are as follows:

(24) $$P=\frac{{\mathrm{Num}}_{ae}}{{\mathrm{Num}}_{at}}$$

(25) $$R=\frac{{\mathrm{Num}}_{aca}}{{\mathrm{Num}}_{vca}}$$

(26) $$F=2\cdot \frac{P\cdot R}{P+R}$$

Here, *Num_ae_* represents the total number of keyframes in all complete assembly line actions extracted by the method, *Num_at_* is the total number of keyframes extracted, *Num_aca_* is the number of complete assembly line actions captured, and *Num_vca_* is the total number of assembly line actions in the video. Precision (*P*) measures the proportion of true keyframes among all extracted keyframes, with higher precision indicating that most of the extracted frames provide valuable information with minimal noise. Recall (*R*) evaluates the proportion of complete assembly line actions captured, with higher recall indicating effective coverage of key actions in the video. The F-score (*F*), as the harmonic mean of precision and recall, offers a comprehensive evaluation of the quality of the keyframes extracted by the method.

(2) Compression Rate (*CR*):

(27) $$CR=\frac{{\mathrm{Num}}_{at}}{{\mathrm{Num}}_{vt}}$$

Num_vt_ represents the total number of frames in the original video. The compression rate reflects the extent to which the method simplifies the original video content by extracting keyframes.

(3) Processing Speed (*PS*):

(28) $$PS=\frac{{\mathrm{Num}}_{vt}}{\mathrm{Time}}$$

*Num_vt_* represents the total number of frames in the input video. Since the execution time of the same method may fluctuate on the same computer, the value of *Time* is the average time taken by the method over 100 runs. *Time* includes the processing time for all steps, such as input video handling, resolution adjustment, and output video generation. Processing speed (*PS*) reflects the efficiency of the method in processing video data and indicates the average number of frames processed per second.

(4) Average Keyframes per Action (*AKPA*):

(29) $$AKPA=\frac{{\mathrm{Num}}_{ae}}{{\mathrm{Num}}_{aca}}$$

The average keyframes per action reflect how many keyframes, on average, are used to represent each complete assembly line action extracted by the method. A larger average keyframes per action value indicates that the extracted assembly line actions are more comprehensively represented.

(5) Recall and Average Keyframes per Action for Different Types of Actions:

(30) $$R_{i}=\frac{{\mathrm{Num}}_{acai}}{{\mathrm{Num}}_{vcai}}$$

(31) $$AKPA_{i}=\frac{{\mathrm{Num}}_{aei}}{{\mathrm{Num}}_{acai}}$$

Here, *i* represents the type number of the assembly line action, *Num_acai_* is the number of type *i* assembly line actions extracted by the method, *Num_vcai_* is the total number of type *i* assembly line actions in the original video, and *Num_aei_* is the total number of keyframes extracted for type *i* assembly line actions by the method. A higher *R_i_* indicates better capture performance for type *i* assembly line actions by the method, while a higher *AKPA_i_* indicates a more complete representation of type *i* assembly line actions by the extracted keyframes.

### 3.4. Performance Evaluation and Discussion

This study compares the proposed method with five existing keyframe extraction techniques. The SIFT method [21] utilizes a SIFT-point distribution histogram (SIFT-PDH) to detect shot boundaries by combining local and global features. It applies an adaptive threshold for boundary detection and selects keyframes based on entropy-derived singular values. The VSUHCM method [20] calculates higher-order color moments, as well as skewness and kurtosis histograms from image blocks, to identify shot boundaries. Keyframes are then selected based on blocks with the highest mean and standard deviation values. The STIMO method [27] uses a fast clustering algorithm based on HSV color distribution to construct both static and dynamic video storyboards. It employs an improved farthest point first (FPF) clustering technique to identify representative frames for summarization. The VSUMM method [28] extracts HSV color histograms from video frames and applies k-means clustering to group similar frames. From each cluster, representative frames are selected as keyframes for static video summarization. Lastly, the ResNet method [25] adopts a deep learning approach. It extracts deep features from video frames using a pre-trained ResNet-151v2 model and then applies k-means clustering to group similar frames. Keyframes are chosen as those closest to the cluster centers, ensuring a compact yet informative summary.

**Figure 10:**
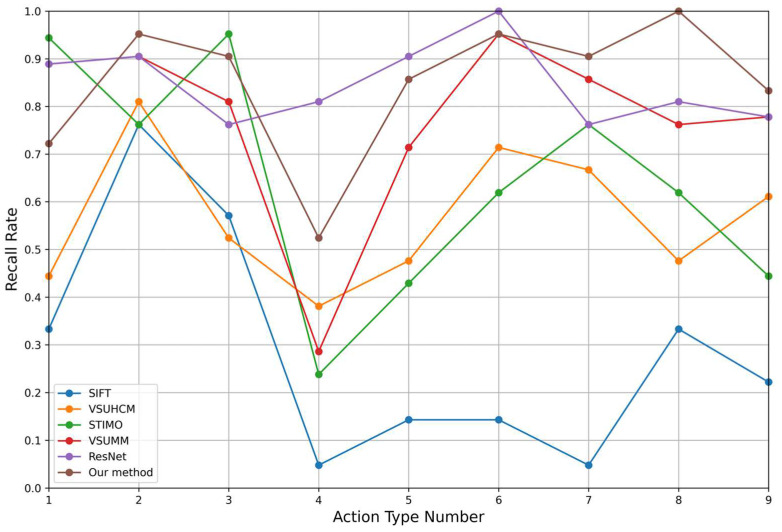
Recall rates of various actions for different methods.

**Figure 11:**
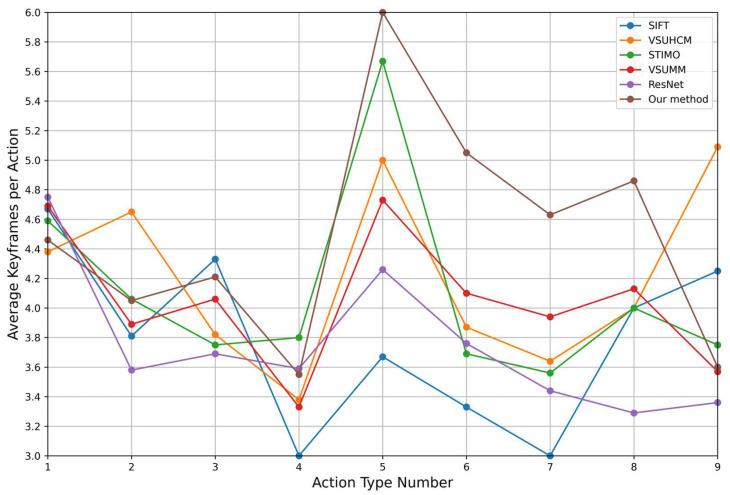
*AKPA* of various actions for different methods.

Table 2 summarizes the performance of each method across six evaluation metrics. Table 3 and Figure 10 present the recall rates for different action categories, while Table 4 and Figure 11 report the Average Keyframes per Action (*AKPA*) achieved by each method across these categories.

From Table 2 and Table 3, it can be observed that, compared with the other five methods, the proposed method ranks first across all six evaluation metrics. Not only is it the fastest in processing video frames, but the quality of the extracted keyframes is also the highest. The proposed method achieves a compression rate of 6.63%, a processing speed of 274.19 frames per second, and a recall rate of 85.2%. These results demonstrate the high performance level of the proposed method.

Additionally, an ablation experiment was conducted to analyze the impact of selecting different clustering algorithms on the performance of the proposed method.

As shown in Table 5, replacing the k-means++ algorithm with the k-means algorithm enhances the method’s ability to capture assembly line actions. However, this modification reduces video frame processing speed by approximately 49%. Given that the proposed method is designed for industrial assembly line scenarios, where efficiency is a priority, k-means++ was ultimately chosen as the clustering algorithm.

**Figure 12:**
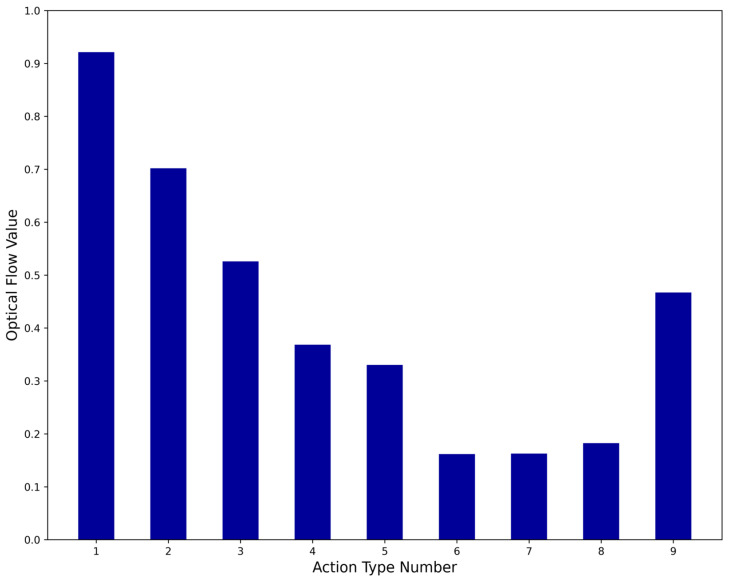
Optical flow values of various pipeline actions.

The optical flow value of a specific action is defined as the sum of the optical flow values of all frames for that action divided by the total number of frames. The optical flow value of a type of assembly line action is defined as the sum of the optical flow values of all actions of that type divided by the number of actions. As shown in Figure 12, the optical flow values for assembly line actions 6, 7, and 8 are relatively small, indicating that these actions have smaller amplitudes. In the proposed method, the recall rates for these three assembly line actions are all above 90%, and the *AKPA* values are all above 4.6. Moreover, the *AKPA* for actions 5, 6, 7, and 8 in the proposed method are greater than those achieved by the other comparison methods. This indicates that the proposed method is more targeted toward small-amplitude actions and demonstrates a stronger capability in capturing small-amplitude assembly line actions compared to the other four methods. The superior performance of the proposed method on assembly line operation videos can be attributed to the following factors:

(1) As shown in Figure 12, the optical flow values of assembly line actions are concentrated between 0.10 and 0.95, and Figure 6 shows that the optical flow values of frames in the assembly line video range from 0.0 to 4.0. This indicates that the frames of assembly line actions generally have smaller optical flow values, meaning assembly line actions are typically small-amplitude actions. The proposed method classifies video frames based on their optical flow values and extracts a higher proportion of keyframes from frames with smaller optical flow values, making it more targeted toward small-amplitude actions. As a result, the method captures more frames related to assembly line actions, achieves stronger action capture capability, and improves the overall quality of the extracted keyframes.

(2) The proposed method groups and clusters video frames based on their sequential numbers and categories. The content of the keyframes in the same group is relatively similar, which enhances the clustering effect. Additionally, it ensures a more uniform distribution of the extracted keyframes across the original video, preventing keyframes from being overly concentrated in specific segments and improving the overall quality of the extracted keyframes.

(3) The proposed method calculates optical flow values using the DIS algorithm and extracts image features using the ORB algorithm. Both algorithms have low time complexity. Furthermore, by constructing a bag-of-visual-words model, the method encodes video frames into feature vectors of only 32 dimensions, reducing the time required for clustering and satisfying real-time processing requirements.

## 4. Conclusions

This paper addresses the challenges of excessive redundancy in assembly line operation videos and the limitations of existing keyframe extraction methods in capturing subtle actions. We propose a novel extraction approach based on optical flow estimation and ORB features, incorporating adaptive strategies tailored to varying motion amplitudes. The method encodes video frames into feature vectors, groups them according to optical flow values and temporal continuity, and applies different compression rates to each group. Keyframes are then extracted via clustering within each group. Compared with several established methods, the proposed approach demonstrates superior processing efficiency and significantly improved performance in identifying small-amplitude actions. It effectively preserves essential content while eliminating redundant frames, making it highly suitable for industrial video analysis. Beyond enhancing operational oversight and reducing management costs, the method introduces a new paradigm in keyframe extraction through motion-aware grouped processing.

## Figures and Tables

**Table 1:** Running environment.

Operating System	Windows 11 Pro
Hardware	CPU: Intel Core i5-13490F
Memory	32 GB
Software	PyCharm 2023.2.5

**Table 2:** Performance comparison of methods.

Method	*P* (%)	*R* (%)	F-Score (%)	*CR* (%)	*PS*	*AKPA*
SIFT [21]	16.9	29.0	21.4	6.74	46.81	4.02
VSUHCM [20]	35.2	56.8	43.5	6.67	137.60	4.21
STIMO [27]	38.4	63.9	48.0	**6.63**	226.26	4.06
VSUMM [28]	46.8	77.0	58.2	**6.63**	153.65	4.10
ResNet [25]	46.9	84.7	60.2	0.66	4.51	3.75
Our method	**57.5**	**85.2**	**68.7**	**6.63**	**274.19**	**4.56**

Bold numbers indicate the best performance in each column.

**Table 3:** Recall rates of different methods for various actions.

Method	Action 1	Action 2	Action 3	Action 4	Action 5	Action 6	Action 7	Action 8	Action 9
SIFT [21]	0.333	0.762	0.571	0.048	0.143	0.143	0.048	0.333	0.222
VSUHCM [20]	0.444	0.810	0.524	0.381	0.476	0.714	0.667	0.476	0.611
STIMO [27]	**0.944**	0.762	**0.952**	0.238	0.429	0.619	0.762	0.619	0.444
VSUMM [28]	0.889	0.905	0.810	0.286	0.714	0.952	0.857	0.762	0.778
ResNet [25]	0.889	0.905	0.762	**0.810**	**0.905**	**1.000**	0.762	0.810	0.778
Our method	0.722	**0.952**	0.905	0.524	0.857	0.952	**0.905**	**1.000**	**0.833**

Bold numbers indicate the best performance in each column.

**Table 4:** *AKPA* of different methods for various actions.

Method	Action 1	Action 2	Action 3	Action 4	Action 5	Action 6	Action 7	Action 8	Action 9
SIFT [21]	4.67	3.81	**4.33**	3.00	3.67	3.33	3.00	4.00	4.25
VSUHCM [20]	4.38	**4.65**	3.82	3.38	5.00	3.87	3.64	4.00	**5.09**
STIMO [27]	4.59	4.06	3.75	**3.80**	5.67	3.69	3.56	4.00	3.75
VSUMM [28]	4.69	3.89	4.06	3.33	4.73	4.10	3.94	4.13	3.57
ResNet [25]	**4.75**	3.58	3.69	3.59	4.26	3.76	3.44	3.29	3.36
Our method	4.46	4.05	4.21	3.55	**6.00**	**5.05**	**4.63**	**4.86**	3.60

Bold numbers indicate the best performance in each column.

**Table 5:** Impact of clustering algorithm selection on method performance.

Clustering Algorithm	*P* (%)	*R* (%)	F-Score (%)	*CR* (%)	*PS*	*AKPA*
k-means	**60.4**	**88.5**	**71.8**	**6.62**	139.75	**4.60**
k-means++	57.5	85.2	68.7	6.63	**274.19**	4.56

Bold numbers indicate the best performance in each column.

## Data Availability

The corresponding author is available to answer any questions.
